# The Impact of Iodinated Contrast Media Used in Epidural Steroid Injections on Thyroid Function Tests

**DOI:** 10.7759/cureus.70888

**Published:** 2024-10-05

**Authors:** Ridvan Isik, Serdar Kokar, Yucel Olgun, Savas Sencan, Hakan Gunduz

**Affiliations:** 1 Division of Pain Medicine, Department of Physical Medicine and Rehabilitation, Sakarya Training and Research Hospital, Sakarya, TUR; 2 Division of Pain Medicine, Department of Physical Medicine and Rehabilitation, Marmara University, Istanbul, TUR

**Keywords:** contrast media, epidural injection, iodine, thyroid functions, thyroid pathology

## Abstract

Objective: The use of iodinated contrast media (ICM) inevitably increases in conjunction with the growing number of epidural steroid injection (ESI) administrations. The purpose of this study was to investigate the relationship between thyroid functions and ICM exposure through ESI procedures.

Design: This is a prospective observational study.

Setting: The study was conducted at a university hospital pain management center.

Methods: This study was conducted between June 2022 and February 2023. Participants between the ages of 18 and 65 who had received an ESI at our outpatient pain clinic comprised the study's population. Thyroid function tests (free triiodothyronine (FT3), free thyroxine (FT4), and thyroid-stimulating hormone (TSH)) were measured before and three weeks after the procedure.

Results: A total of 124 participants (80 women and 44 men) were analyzed. The average amount of contrast media administered was 1.34 ml. A significant increase was observed only in the FT4 value compared to pre-procedure (p = 0.017), and no patient developed thyroid disorders.

Conclusion: The current study presents an analysis of the relationship between ICM and thyroid functions in the ESI population.

## Introduction

Spinal pain management has become a major health issue as a result of longer life expectancies and an increase in the frequency of age-related spinal disorders. The current clinical treatment of spinal conditions is composed of nonsteroidal anti-inflammatory drugs (NSAIDs), physical therapy, therapeutic injections, and surgery.

Epidural steroid injections (ESIs) are one of the most often used procedures for managing spinal pain among the various treatment modalities that are available [1]. There have been reports of substantial increases in ESI utilization among the US Medicare population, reflecting a 24% annual growth rate [2]. ESI can be implemented precisely and securely with the use of computed tomography (CT) or fluoroscopy. Even so, in order to confirm the exact needle tip position and avoid inadvertent non-epidural injections (intravascular, subdural, subarachnoid, etc.), the use of iodinated contrast media (ICM) is crucial and imperative [3]. 

The amount of contrast used in ESIs varies depending on factors such as procedure type, number of procedures, practitioner experience, and anatomical structures. Since many factors are involved in the procedures, even though a small dosage of contrast media is generally adequate, these doses may be raised, which might also elevate the risk of the contrast media's adverse effects.

The amount of iodine in ICM is considerable. The amount of iodine that the patient may receive during a single CT scan might range from 100 to thousands of times the normal limit [4,5]. Free iodine in the serum is utilized by the thyroid gland to synthesize thyroid hormone for metabolic processes. The normal thyroid gland is capable of adapting to an increase in iodine. When rapid overload iodine doses are administered suddenly, the regulation of thyroid hormone is disrupted, which can lead to either hyperthyroidism (the Wolff-Chaikoff effect) or hypothyroidism (the Jod-Basedow effect) [6-8].

The impact of ICM on nephrotoxicity has been widely explored; however, several studies have addressed ICM's effects on the normal thyroid gland in clinical practice, and most of those studies were related to the use of CT scans [9,10]. To the best of our knowledge, there have been no reports examining the association between ICM exposure and ESI procedures applied with fluoroscopy guidance and subsequent thyroid functionality. We hypothesize that ICM used in ESIs may compromise thyroid gland functions and the dysfunction of thyroid hormones may be correlated with ICM's dose.

Hence, the aim of this prospective research is to determine whether exposure to ICM is linked to thyroid dysfunction in the ESI population.

The manuscript is presented as a preprint at https://www.researchsquare.com/article/rs-3401473/v1 on October 16, 2023.

## Materials and methods

**Figure 1:**
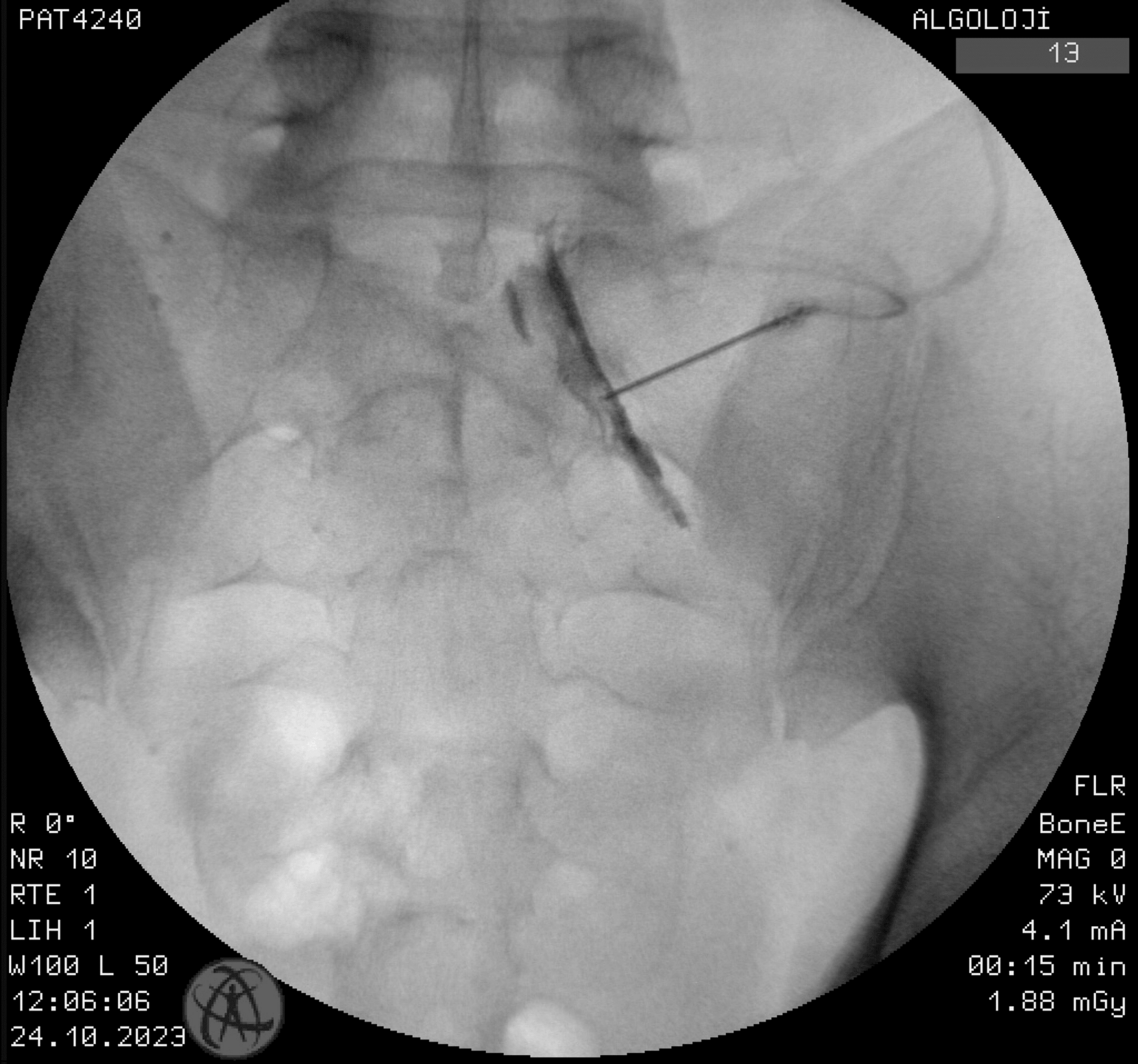
Contrast media spreading during lumbar transforaminal epidural injection

**Figure 2:**
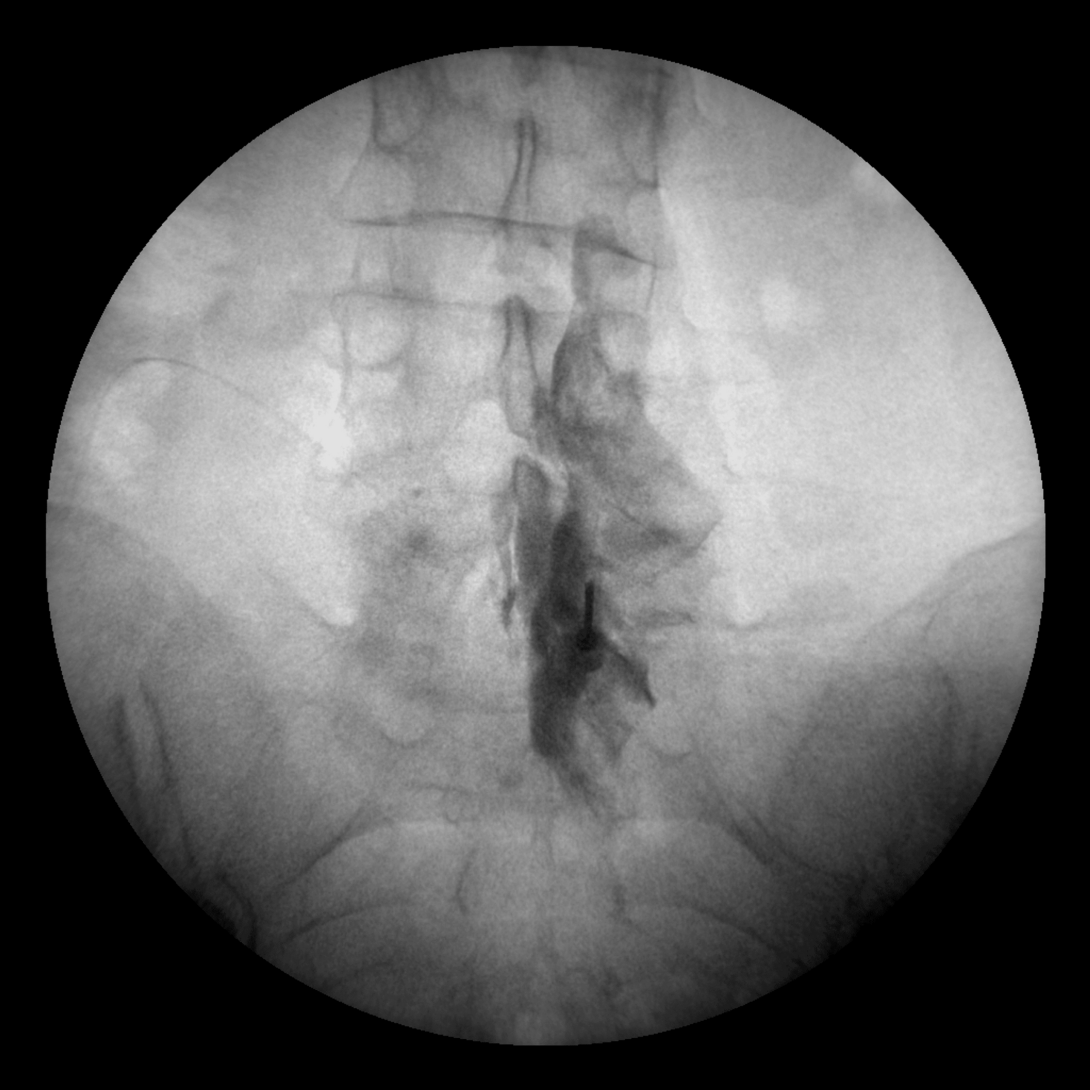
Contrast media spreading during lumbar interlaminar epidural injection

**Figure 3:**
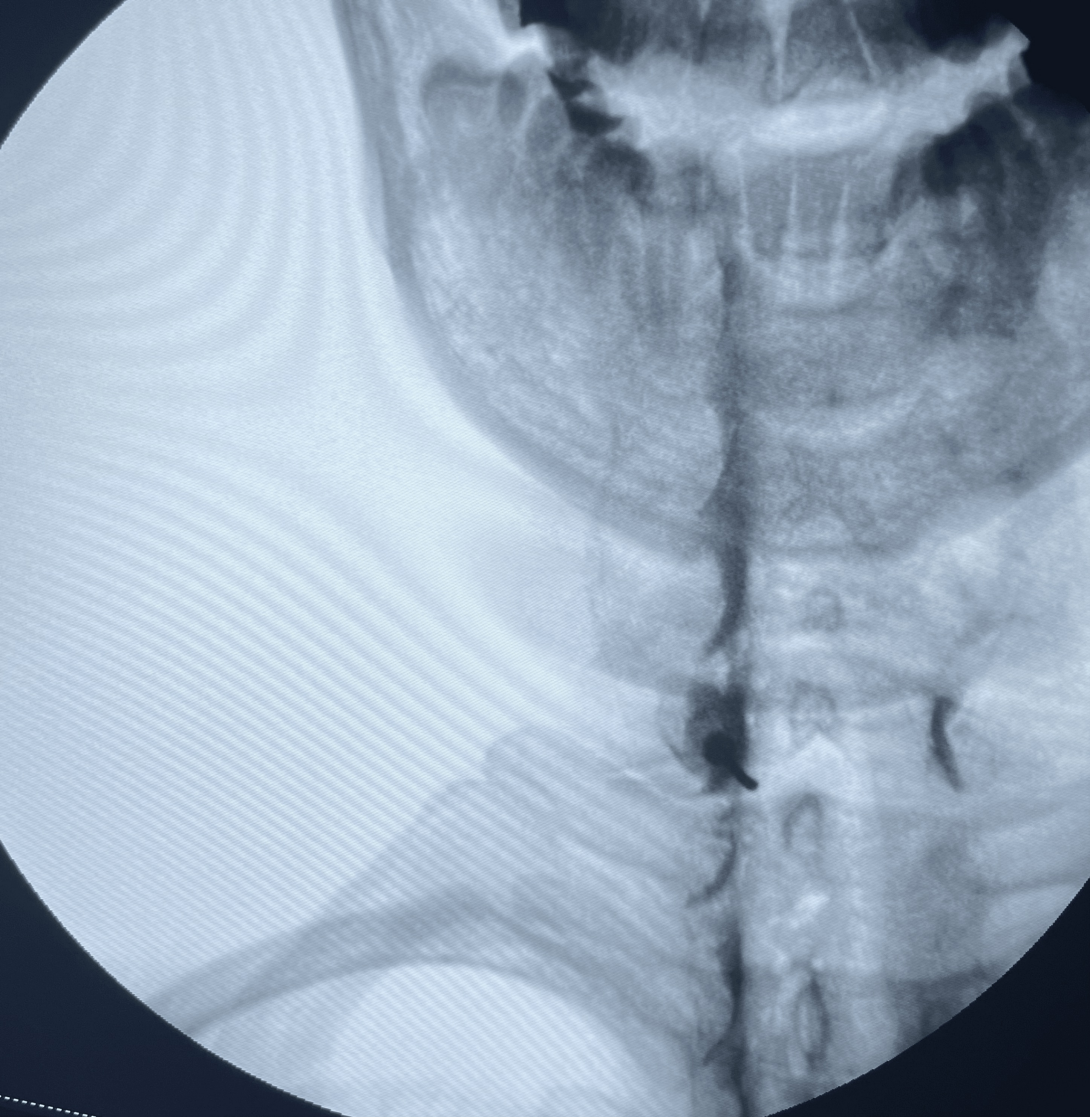
Contrast media spreading during cervical interlaminar epidural injection

This prospective observational study was carried out at Marmara University Pendik Training and Research Hospital outpatient pain clinic from June 2022 to February 2023. The study was approved by the Marmara University School of Medicine Ethics Committee (approval number: 07.01.2022.82) and conducted in accordance with the principles of the Declaration of Helsinki. The study was registered at ClinicalTrials.gov (NCT05404412). The population of the study consisted of patients between the ages of 18 and 65, who had undergone any type of ESI, including lumbar, cervical, and caudal ESIs, for any reason in our outpatient clinic (Figure 1, Figure 2, and Figure 3). 

Exclusion criteria were as follows: (A) suffering or had suffered thyroid or metabolic diseases, thyroid dysfunction, or thyroid surgery; (B) suffering or had suffered hypothalamic or pituitary diseases; (C) a current prescription for levothyroxine, antithyroid drugs, amiodarone, or lithium; (D) systemic and/or local infections, malignancy, or bleeding diathesis; (E) a known allergy to contrast material and/or local anesthetic substances; (F) a known history of any psychiatric disorder; and (G) a history of pregnancy and pregnant women. Verbal and written informed consent was obtained from all patients participating in the study.

**Figure 4:**
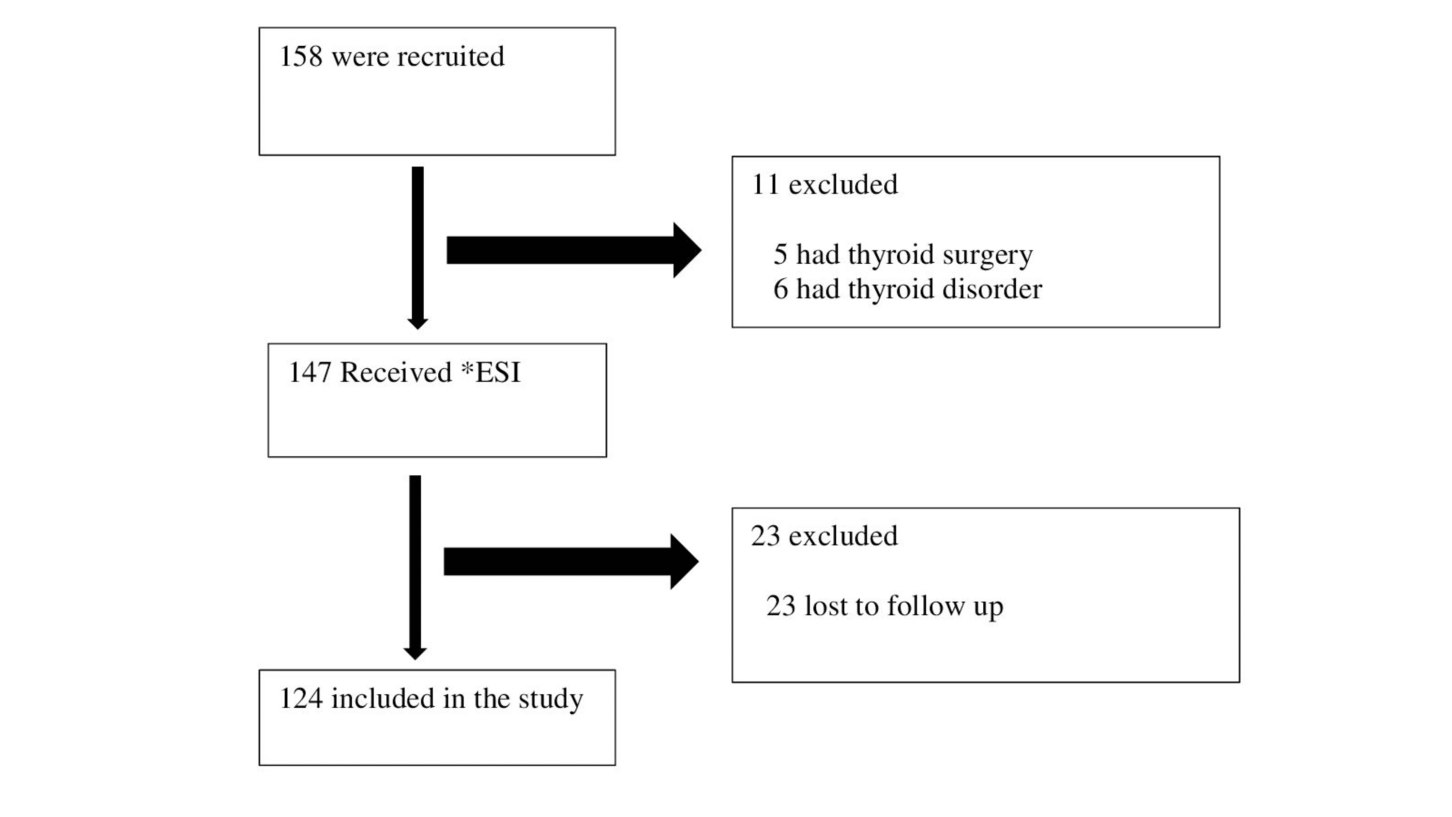
Flowchart of the study *ESI: epidural steroid injection

The flowchart for determining the study population is displayed in Figure 4.

In conjunction with the recording of the demographic data of the patients participating in this study (age, sex, body mass index (BMI), contrast medium doses, etc.), the values of thyroid function tests (TFTs) including free triiodothyronine (FT3), free thyroxine (FT4), and thyroid-stimulating hormone (TSH) were analyzed before the procedure and the third week after the procedure. The Immuno-Serology Laboratory measured serum TSH, FT3, and FT4. Blood samples were collected from the participants at the same hours of the day before and after the procedure while fasting.

TSH, FT4, and FT3 were measured using electrochemiluminescence immunoassay (Cobas Integra 8000, Roche Diagnostics, Germany). The reference ranges for TSH, FT3, and FT4 were 0.48-4.81 mIU/L, 2.02-4.42 ng/L, and 0.78-1.51 ng/dL, respectively.

The laboratory analyses have been authorized for use in clinical settings. ESIs were performed by a 10-year experienced interventional pain specialist (SS) under sterile conditions with the C-arm fluoroscopy guidance. Throughout the process, details on the procedure that can affect the outcomes were recorded, including the type of ESI, the number of interventions, the frequency of inadvertent injections, the volume of the contrast, and the route of the contrast administration.

The total contrast volume was measured manually after the procedure. The same injector type was used throughout all procedures. All patients were given the low-osmolar, nonionic contrast medium iohexol (Omnipaque, 300 mg iodine/mL, GE HealthCare, Chicago, IL, USA) for ESIs.

## Results

A total of 124 participants who had received an ESI were analyzed. Forty-four (35.5%) of the patients were male, and 80 (64.5%) were female. While the mean age of the patients was 49.32 ± 9.70, the BMI was 28.38 ± 4.46. Of the 124 procedures performed, 99 (79.8%) were lumbar, 21 (16.9%) were cervical, and four (3.2%) were caudal ESIs.After contrast agent administration, thyroid functions have not deteriorated, and all patients were euthyroid (Table 1).

**Table 1:** Demographic data, procedure types, and post-procedure thyroid function The descriptive data was provided as n (%), mean ± SD (min-max), and median (min-max) values. ESI: epidural steroid injection; BMI: body mass index

Variable values	Mean ± SD
Age (years)	49.32 ± 9.70
BMI (kg/m^2^)	28.38 ± 4.46
The amount of contrast media (ml)	1.34 ± 0.63
Gender	n (%)
Male	44 (35.5)
Female	80 (64.5)
Procedure	n (%)
Lumbar ESI	99 (79.8)
Cervical ESI	21 (16.9)
Caudal ESI	4 (3.2)
Post-procedure thyroid function	n (%)
Euthyroidism	124 (100)

There was a significant increase in FT4 post-contrast injection related to the FT4 measurement before injection (p = 0.017). In contrast, no significant changes were detected in serum TSH (p = 0.810) and serum FT3 (p = 0.972) (Table 2). No major adverse events were observed during the procedures. As a result of the correlation analysis performed between the contrast agent doses and TFTs, no strong correlation was found (Table 3 and Table 4).

**Table 2:** Comparison of TFT values before and after contrast administration Statistical analyses were conducted using the paired t-test for T3 and T4 and the Wilcoxon test for TSH. TSH: thyroid-stimulating hormone; TFT: thyroid function test; T3: triiodothyronine; T4: thyroxine

TFTs	Pre-procedure	Post-procedure	P-value
T3	2.98 ± 0.44	2.98 ± 0.53	0.972
T4	1.21 ± 0.24	1.25 ± 0.24	0.017
TSH	1.59 (0.13-4.72)	1.54 (0.10-9.83)	0.810

**Table 3:** Correlation between contrast media and T3-T4 values *Correlation is significant at the 0.05 level. T3: triiodothyronine; T4: thyroxine

-	Contrast media		preT3	preT4	postT3	postT4
Contrast media	r	1	0.194^*^	0.002	0.025	-0.149
	p	-	0.038	0.982	0.785	0.099
preT3	r	-	1	-0.090	0.441^*^	-0.109
	p	-	-	0.341	<0.001	0.247
preT4	r	-	-	1	-0.066	0.724^*^
	p	-	-	-	0.481	<0.001
postT3	r	-	-	-	1	0.090
	p	-	-	-	-	0.331
postT4	r	-	-	-	-	1

**Table 4:** Correlation between contrast media and TSH values *Correlation is significant at the 0.05 level. TSH: thyroid-stimulating hormone

-		Contrast media	preTSH	postTSH	
Contrast media	rho	1	0.135	0.059	
	p	-	0.136	0.518	
preTSH	rho	-	1	0.667^*^	
	p	-	-	<0.001	
preTSH	rho	-	-	1	

## Discussion

This prospective observational study was carried out to evaluate the impact of contrast media on thyroid function in patients who received ESI. First, we revealed that the serum level of FT4 was significantly elevated but still remained in normal ranges following ESI. Second, none of our patients developed thyroid dysfunction. Third, we discovered that these conditions were unrelated to the ICM dosage.

ICM is routinely and necessarily employed in ESI procedures. An average 1.5-2 ml dosage of ICM with a 35 μg/ml concentration is used in the procedure, providing 70 μg of free iodide, which is much lower than CT or angiography ranges [11,12]. Some research indicates that repeated ICM exposure and large ICM volumes are more likely to elevate the risk of thyroid disorders [13,14]. Contrary to these studies, we found no correlation between contrast volume and thyroid hormone dysregulation. This might be due to the low amount of contrast media and the short follow-up period.

The iodine or iodide load provided by ICM likely explains the observed connection between ICM exposure and hyperthyroidism. Once the iodine has entered the thyroid follicles, it is used to produce the thyroid hormones T4 and T3 [15]. After an iodine dose, defective autoregulation is the mechanism behind ICM-induced hyperthyroidism, and a high consumption of iodine will lead to either temporary or permanent hyperthyroidism [16-18]. This mechanism may shed light on the link between ICM exposure and thyroid function changes in the studies. Similar to the findings of Chen et al., we discovered a slight difference in T3 and TSH levels but a significant change in T4 levels compared to pre-treatment. However, when compared, the amount of contrast material used differs [10]. We postulated that this may be due to non-dose-dependent personal and other variables, including living in an iodine-deficient region and having an underlying autoimmune disease or thyroid issues (euthyroid goiter, euthyroid sick syndrome, etc.).

None of the participants in our research experienced any thyroid function deterioration after ICM exposure. A prospective observational cohort research examined the long-term consequences on thyroid function in euthyroid individuals employing ICM for coronary angiography. After eight weeks, they discovered that ICM may give rise to subclinical hyperthyroidism [19]. It has been proposed that the association between ICM exposure and developing thyroid dysfunction may be more widespread than previously anticipated [20]. A nested case-control study between 1990 and 2010 consisting of 3678 individuals was conducted in the euthyroid population by Rhee et al. [6]. They showed that overt hypothyroidism and hyperthyroidism were significantly associated with ICM exposure. Over the course of a year, one million patients in the general population of Taiwan showed a considerably greater incidence of ICM-induced thyroid dysfunction after six years of observation [5]. Using Taiwan's National Health Insurance Research Database, a later investigation discovered that individuals with euthyroid nodular goiter had a nearly fivefold greater incidence of ICM-induced thyroid dysfunction than those without thyroid nodules [21]. Yet, in certain studies, ICM exposure caused thyroid dysfunction, particularly in patients with a history of thyroid illness or renal insufficiency [14,21,22]. However, since we were unable to recruit adequate patients with thyroid dysfunction, our investigation focused on participants with normal thyroid function. In our subsequent investigation, we intend to include individuals with thyroid disorders.

Despite convincing retrospective research, the outcomes of prospective observational studies in various regions assessing changes in thyroid hormone levels after a single exposure to ICM are highly diverse [23-25]. In accordance with a recent systematic review and meta-analysis, during radiographic processes, the frequency of thyroid dysfunction following ICM injection is remarkably low [23]. These differences may be caused by the selection of participants (based on thyroid functions), the amount and type of contrast agent used, and the follow-up period, which cause substantial heterogeneity among studies [26].

Our study has several limitations. First, our study has a short follow-up period. On the other hand, the relationship between ICM volume and the long-term risk of thyroid illness, as well as the requirement of thyroid function monitoring, remains obscure. Second, due to the nature of the technique, we carried out ESI utilizing low-dose contrast volumes. Third, participants in our study were not strictly selected from an iodine-sufficient region. Fourth, contrast media absorption may vary in different epidural regions; therefore, subgroup analysis may be required. Finally, due to the difficulties in diagnosing asymptomatic autoimmune diseases and goiter, this population may be mistakenly included. It might affect our clinical results.

We also have strengths in our study. It is the first study to address the change in TFTs due to ICM exposure in ESIs.

We demonstrated that even though there is a substantial elevation of T4 level, yet within the normal limit, those procedures could not deteriorate thyroid function values and lead to a thyroid disorder in euthyroid individuals. Nevertheless, interventional pain specialists should keep in mind that the contrast medium used during procedures may worsen this condition in patients with thyroid hormone disorders. Given the growing prevalence of ESI implementations in clinical practice, the results obtained are of importance in the daily routine practice of ESI.

## Conclusions

The current study provides an overview of the association between ICM and thyroid functions in the ESI population. In this study, there was no meaningful difference in TSH and T3 values before and after injection, whereas a significant increase in T4 value was observed in post-injection individuals, but still remained within normal limits. Given the widespread use of contrast media in interventional pain management such as ESI, routine thyroid examinations prior to and after ESI are required, particularly for patients who are inclined to thyroid dysfunction. Consequently, physicians and patients should be acquainted with the possible thyroid issues related to ICM, and in order to clarify this issue, further studies with various contrast media volumes and longer follow-up periods are needed.
